# Association of the Protective Dietary Pattern for Blood Pressure with Elevated Blood Pressure and Hypertension among Chinese Children and Adolescents Aged 6–17 Years Old: Data from China Nutrition and Health Surveillance (2015–2017)

**DOI:** 10.3390/nu15234927

**Published:** 2023-11-26

**Authors:** Yuge Li, Yuxiang Yang, Lahong Ju, Wei Piao, Xiaoqi Wei, Liyun Zhao, Dongmei Yu

**Affiliations:** NHC Key Laboratory of Trace Element Nutrition, National Institute for Nutrition and Health, Chinese Center for Disease Control and Prevention, Beijing 100050, China; liyuge1122@163.com (Y.L.); yxyang_ninhccdc@126.com (Y.Y.); julh@ninh.chinacdc.cn (L.J.); piaowei@ninh.chinacdc.cn (W.P.); weixq@ninh.chinacdc.cn (X.W.); zhaoly@ninh.chinacdc.cn (L.Z.)

**Keywords:** blood pressure, dietary pattern, reduced rank regression, DASH diet, children and adolescents

## Abstract

Studies focused on the association between dietary patterns and elevated blood pressure (BP) and hypertension (HTN) among children and adolescents remain insufficient. This study aimed to explore a dietary pattern that could be helpful for the prevention of abnormal BP and to investigate the association between this dietary pattern and elevated BP and HTN among Chinese children and adolescents. A total of 52,080 Chinese children and adolescents aged 6~17 years old from the China Nutrition and Health Surveillance (CNHS) 2015–2017 were included in the current study. The reduced rank regression (RRR) method was applied to derive a dietary pattern that is associated with BP. Multivariable logistic regression was used to assess the association between dietary pattern (DP) and elevated BP and HTN. The Dietary Approach to Stop Hypertension (DASH) score was also calculated for each participant as a comparative method to validate the dietary pattern derived with the RRR method. A protective dietary pattern (PDP) for BP among Chinese children and adolescents was derived, which was characterized by high intakes of dairy products, mushrooms/edible fungi, fresh vegetables, fresh fruits, fresh eggs, aquatic products, mixed legumes, soybeans and related products, offal, dried fruits, and coarse cereals, with low intakes of refined grains. After multiple adjustments, there were significant inverse associations between PDP scores and the odds of elevated BP and HTN (elevated BP: Q5 vs. Q1, OR = 0.849, 95%CI = 0.755–0.931, P-trend < 0.05; HTN: Q5 vs. Q1, OR = 0.795, 95%CI = 0.694–0.911, P-trend < 0.05). The DASH diet was also observed to have protective effects on elevated BP in model I (Q5 vs. Q1, OR = 0.899, 95%CI = 0.828–0.975, P-trend < 0.05), but was not observed to have protective effects on HTN (HTN: Q5 vs. Q1, OR = 0.958, 95%CI = 0.876–1.048, P-trend > 0.05). The current study suggested that greater adherence to the PDP for BP among Chinese children and adolescents might be associated with lower odds of elevated BP and HTN.

## 1. Introduction

Hypertension (HTN) is a substantial public health issue in China and worldwide [1,2]. However, it is not merely an adult disease. Several studies demonstrated the evidence of blood pressure (BP) tracking from childhood into adulthood [3,4]. An increasing amount of research suggests that elevated BP or HTN in childhood is closely associated with cardiovascular disease (CVD) in adulthood [5,6]. Din-Dzietham et al. reported the increasing pediatric BP trends over the last half-century [7]. In China, childhood HTN has also emerged as a major public health challenge. Ma et al. reported that the prevalence of HTN among Chinese children and adolescents exceeded 20% in 2015 based on the 2018 Chinese Hypertension Prevention and Treatment Guideline reference [8], and that both BP levels and prevalence of HTN increased significantly in Chinese children and adolescents from 1991 to 2015 [9].

Given the rising prevalence of HTN and its adverse outcome for both short and long-term health, greater emphasis should be put on the primary prevention of elevated BP and HTN in Chinese children and adolescents [10]. The contributing factors for HTN include genetic factors, overweight status and obesity, dietary factors, and lifestyle factors, among which diet intervention is one of the main target factors for interventions that aim at controlling and preventing rising BP, with relatively higher cost-effectiveness [11]. Thus, dietary pattern, as an assessment method of the overall diet, has become a helpful approach in studying the association between diet and BP.

Dietary patterns can be assessed using a priori approaches, for example, the Dietary Approaches to Stop Hypertension (DASH) diet which has been shown to have strong associations with hypertension in childhood [12,13]. Dietary patterns can also be derived using a posteriori approaches, for example, principal component analysis (PCA) and factor analysis, which is based on variation in dietary intake of the study population [14]. Reduced Rank Regression (RRR) is a hybrid approach to construct dietary patterns based on the variation in specific markers related to disease by maximally explaining variation in responses (disease-related nutrients) [15]. Therefore, compared to patterns derived from traditional priori methods or posteriori methods, dietary patterns obtained using RRR tend to be more associated with target disease outcomes. In adults, there have been several studies revealing that the dietary pattern derived from RRR was associated with the target disease [16,17,18,19]. Nevertheless, studies on dietary patterns in relation to elevated BP or HTN among children and adolescents using the RRR method are still scarce.

The objectives of the present research are to identify a dietary pattern closely associated with elevated BP and HTN risk among Chinese children and adolescents, and meanwhile, to explore the associations of current dietary pattern scores and DASH scores with elevated BP and HTN, respectively, based on the data from the China Nutrition and Health Surveillance (CNHS) 2015–2017.

## 2. Materials and Methods

### 2.1. Study Population

Data were drawn from the China Nutrition and Health Surveillance (CNHS) 2015–2017, in which CNHS of Children and Lactating Mothers was conducted from 2016 to 2017. As a nationally representative cross-sectional study, the main aim of this study was to obtain the dietary and nutritional information, physical condition, and non-communicable disease condition of participants from 275 survey sites in 31 provinces/municipalities/autonomous in China mainland. Details concerning the study protocol, sampling design, methods, and quality control process have been detailed in our previous study [20]. Participants were excluded based on the following: (1) participants aged over 18 years old or under 6 years old; (2) participants with unavailable basic information (i.e., socioeconomic and lifestyle); (3) participants with invalid or missing physical examination (i.e., BP measurement); (4) participants with invalid or missing dietary survey information; (5) participants with abnormal energy or nutrient intake. A total of 52,080 children and adolescents aged 6–17 years old were included in the final analysis.

The CNHS project was approved by the Ethical Committee of China CDC. Ethical approval numbers were 201519 (date of approval: 15 June 2015) for China Adult Chronic Disease and Nutrition Surveillance (2015) and 201614 (date of approval: 3 June 2016) for China Nutrition and Health Surveillance of Children and Lactating Women (2016–2017), respectively. Informed consent was signed by all participants before the survey.

### 2.2. Basic Information Collection

Well-trained interviewers from the local Center for Disease Control and Prevention (CDC) collected the information on the sociodemographic characteristics and lifestyles of participants using standardized questionnaires. Sociodemographic information included age, gender, living area, geographic region, maternal education, household income, family history of HTN, etc. Lifestyles information included physical activity, sleep duration, sedentary behavior, second-hand smoke exposure, etc.

### 2.3. Anthropometric Measurements

All anthropometric measurements, including height, weight, and BP, were conducted by well-trained staff using unified methods. The height of the children and adolescents was measured using a TZG stadiometer in centimeters (cm) with an accuracy of 0.1 cm. Weight without heavy clothing and footwear was measured with a weighing scale (G&G TC-200k) in kilograms (kg) with an accuracy of 0.1 kg. Body mass index (BMI) was calculated as kg/m^2^. BP was measured by qualified staff according to a standardized procedure. First, children and adolescents were seated at a table and were given 5 min to rest. After the resting period, BP was measured three times with 1 min between each measurement in a sitting position. BP was measured on the left arm, positioned at heart level, using the automatic oscillometric method, using an Omron HBP1300. Eventually, the average BP was calculated for the three measurements for systolic blood pressure (SBP) and diastolic blood pressure (DBP).

### 2.4. Definitions of Blood Pressure Outcomes

The 2017 updated blood pressure references for Chinese children and adolescents aged 3~17 years old were used to define normal BP, elevated BP, and HTN [8]. Age-, sex-, and height-specific SBP and DBP percentile tables were used for defining BP categories. Normal BP was defined as SBP and DBP of less than the 90th percentile; elevated BP was defined as SBP and/or DBP from the 90th to less than the 95th percentile (or SBP/DBP ≥ 120/80 mm Hg); and HTN was defined as SBP and/or DBP of at least the 95th percentile for sex, age, and height, respectively.

### 2.5. Dietary Assessment

Children and adolescents and/or their parents/caregivers were asked to complete a validated 57-item Food Frequency Questionnaire (FFQ) about dietary habits. Consumption frequency and amount of every food item over the past 30 days was reported by participants [20]. Frequency options included the daily/weekly/monthly consuming frequency for all food items. The consuming amount was assessed in grams or milliliters. Pictorials were used to help participants to improve the accuracy and efficiency of food intake estimation. In the current study, the above 57 items were integrated into 24 food groups (Supplemental Table S1). The daily dietary amount of all food items was aggregated based on consumption frequency and weight during the past 30 days. The daily energy and nutrient intake of individuals were calculated based on the China Food Composition Table (2018). Edible oil and other condiments were not measured with the FFQ used in the CNHS, so we did not include this part in the current study. Nutrient intake data did not include the consumption of supplements and/or medications.

### 2.6. Dietary Pattern Analysis

RRR is a recently emerging statistical method used to derive DPs. It first selects response variables (disease-related nutrients) based on prior knowledge as the intermediate variables, and then constructs linear functions of predictors (foods) by maximally explaining variation in response variables (nutrients) to examine the mechanistic pathway from dietary factors to target outcomes [15]. Therefore, compared to patterns derived from traditional priori methods, such as DASH scores, or posteriori methods (i.e., principal component analysis and factor analysis), dietary patterns obtained using RRR tend to be more associated with disease outcomes [15].

In the current study, RRR was applied to extract dietary patterns from 24 food groups, namely, explanatory variables. Potential response variables for RRR that are known to be related to BP were identified based on previously published studies. Finally, five HTN-related nutrients, including dietary fiber, calcium, potassium, phosphorus, and retinol, were specified as response variables [21,22,23,24,25,26,27,28,29]. In this study, the first pattern was kept for subsequent analysis, which explained the largest variance in all response variables. Factor loadings, which represent the correlation between the factors and food groups, of each food group were also determined. Higher absolute factor loadings reflect higher consumption. Food groups with absolute factor loadings >0.1 were used to describe the dietary pattern, but all food groups contributed to calculating the dietary pattern score. The dietary pattern score was calculated by summing the product of standardized energy-adjustment (intake unit/1000 kcal) intakes of 24 food groups and their corresponding factor loadings. Then, participants were classified into five groups by quintiles of dietary pattern scores, on which the highest quintile portrays a high intake.

Furthermore, the Dietary Approaches to Stop Hypertension (DASH) would be used as a comparative method to assess the validity of the extracted dietary pattern [30]. Food components evaluated included: (1) fruits (including 100% fruit juices), (2) vegetables (except mixed legumes), (3) nuts and seeds, (4) whole grains, (5) dairy products, (6) sodium, (7) red and processed meats, and (8) sweetened beverages. To neutralize the effect of energy intakes on the eight components of the DASH score, the DASH dietary pattern score was calculated by summing the points of intake of 8 food groups. The first five groups were considered protective and the last three were considered a health risk. For the protective food groups, they were scored on a scale from 1 to 5. This means that if the intake of a child was ranked in quintile 5, it was awarded with 5 points, and if the child was ranked in quintile 1, it was awarded with 1 point. For the unhealthy food components, the scoring was reversed. Thus, the total DP scores range from 8 to 40, in which a high score is a high adherence to a DASH diet and a low score is a low adherence to the DASH diet. Regarding sodium intake, sodium from foods was calculated according to the food composition tables used. Sodium from condiments was not included in the current study.

### 2.7. Covariates

The covariates in the sample description and logistic regression analysis were as follows: (1) The age group was divided into 6~11 years old and 12~17 years old. (2) According to the BMI, the participants were classed as normal, overweight, and obese based on age- and sex-specific percentile tables from the recommendations of the Working Group on obesity in China [31]. (3) The living area was categorized as urban areas or rural areas. (4) The geographic region was categorized as east, central, and west regions. (5) The maternal education level was grouped as primary school and below, junior middle school, or senior high school and above. (6) The household income level was divided into not given, low (<10,000 CNY), medium (10,000–25,000 CNY), or high (>25,000 CNY), based on the annual household income per capita. (7) The family history of HTN (No/Yes) was defined as any one of the lineal relatives (including grandparents, parents, or siblings) who had been diagnosed with hypertension. (8) The second-hand smoking exposure status was divided into No/Yes. (9) The sleep duration was divided into adequate and inadequate according to the criteria of American Academy of Sleep Medicine (AASM) [32]. (10) The physical activity was divided into adequate (moderate to vigorous physical activity, MVPA, ≥60 min per day on average), and inadequate (MVPA < 60 min per day on average) according to the recommendation of the Physical Activity Guidelines for Chinese (2021) [33]. (11) The sedentary behavior was grouped as <2 h, 2~3 h, or ≥4 h per day. 

### 2.8. Statistical Analysis

PROC PLS process in SAS software was applied to extract the dietary pattern and calculate dietary pattern scores. Distributions of demographic and lifestyle behavior characteristics were described based on quintiles of dietary pattern scores. Categorical variables were described with the number and related proportion. The Chi-square test and Cochran–Armitage test for categorical variables tested statistical differences and linear trends. Continuous variables were summarized by the median (P25, P75). The Jonckheere–Terpstra test for continuous variables tested linear trends. Spearman rank correlation analysis was used to assess the correlation between explanatory variables (absolute factor loadings ≥ 0.1), response variables, and dietary pattern scores.

Multivariate logistic regression models were used to estimate the odds ratios (ORs) and 95% confidence intervals (CIs) for each quintile of dietary pattern scores in relation to the risk of elevated BP and HTN, taking the first quintile (the lowest) as the reference. The crude model results for elevated blood pressure and hypertension are left blank in the table. Model I was adjusted for gender, age, and BMI, and model II was further adjusted for living areas, geographic regions, maternal education, household income, physical activity, sedentary behavior, sleeping time, family history of HTN, second-hand smoking exposure, daily sodium intake from foods (mg/d), and daily energy intake (kcal/d). P-trend was calculated using the median value of each quintile as a quasi-continuous variable in the model.

Subgroup analysis was performed to assess whether the association of DP and HTN varied by gender (male and female), age (6~11 years old and 12~17 years old), BMI (normal, overweight, and obesity), living areas (urban and rural), maternal education level (primary school and below, junior middle school, and senior high school and above), physical activity (adequate and inadequate), family history of HTN (No/Yes), and second-hand smoke exposure (No/Yes). Effect modification was also detected by adding interaction terms of the modifier and quintiles of DP scores in the full-adjustment logistic model. 

*p* < 0.05 was the threshold for statistical significance. Statistical analyses and plot drawings were conducted with SAS version 9.4 (SAS Institute Inc., Cary, NC, USA) and R version 4.2.2.

## 3. Results

### 3.1. Protective Dietary Pattern for Children and Adolescents Blood Pressure

**Figure 1:**
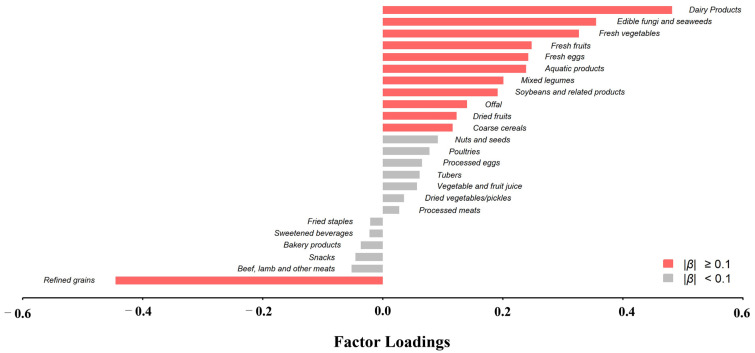
Factor loadings of 24 food groups in the protective dietary pattern for children’s and adolescents’ blood pressure.

The protective dietary pattern (PDP) for children’s and adolescents’ blood pressure derived using RRR explained 47.4% of the response variation and was kept in the subsequent analyses. As shown in Figure 1, this protective dietary pattern for children’s and adolescents’ blood pressure was characterized by high intakes of dairy products, mushrooms/edible fungi, fresh vegetables, fresh fruits, fresh eggs, aquatic products, legumes, soybeans and related products, offal, dried fruits, and coarse cereals, with low intakes of refined grains. For the individual response variable, the explained variation ranged from 25.0% (for retinol) to 53.3% (for potassium). The corresponding food items and factor loadings of different food groups are shown in Supplemental Table S1. As shown in Supplemental Table S2, the dietary score had a positive correlation with all individual response variables.

### 3.2. Characteristics of Study Participants

A total of 52,080 children and adolescents aged 6~17 years old (50.1% males and 49.9% females) were included in the analysis. The participants’ characteristics by quintiles of dietary pattern scores are presented in Table 1. Compared with the lowest quintile, children and adolescents with the highest dietary pattern score tended to be girls, younger, overweight or obesity, from urban or East China, with more highly educated mothers, higher household income levels, less physically active, more sedentary behavior, less sleep duration, and more family history of HTN (all *p* < 0.0001). Furthermore, the highest quintile of dietary pattern scores had more children and adolescents who were exposed to second-hand smoke (*p* = 0.0182).

### 3.3. Food and Nutrients Daily Intake According to Quintiles of PDP Scores for Children’s and Adolescents’ Blood Pressure

Children and adolescents with higher PDP scores consumed more food with positive factor loadings, and, meanwhile, they consumed less refined grains with a negative factor loading. Although the median intake of some food groups (mixed legumes, processed eggs, vegetable and fruit juice, dried vegetables/pickles, and fried staples) was equal to 0 in each quintile, significant differences were observed (all *P*-trend < 0.0001). For energy and nutrient intake (per 1000 kcal), children and adolescents with higher dietary pattern scores had higher intakes of all the nutrients, except for carbohydrates. Further information is available in Supplemental Tables S3 and S4.

### 3.4. Association of PDP and DASH Scores with Blood Pressure Outcomes

Table 2 provides the association between PDP for children’s and adolescents’ blood pressure identified with RRR and DASH scores and the risk of elevated BP and HTN, respectively. For the PDP, the scores were negatively associated with the risk of elevated BP and HTN in a dose–response manner (*P*-trend < 0.001) in model I. In the fully adjusted model, the results remained stable (elevated BP: Q5 vs. Q1, OR = 0.849, 95%CI = 0.755–0.931, *P*-trend = 0.0114; HTN: Q5 vs. Q1, OR = 0.795, 95%CI = 0.694–0.911, *P*-trend = 0.0018).

The DASH diet was also observed to have protective effects on elevated BP and HTN in model I (elevated BP: Q5 vs. Q1, OR = 0.899, 95%CI = 0.828–0.975, *P*-trend = 0.0055; HTN: Q5 vs. Q1, OR = 0.861, 95%CI = 0.769–0.964, *P*-trend = 0.0135), but was not observed to have protective effects in the fully adjusted model (elevated BP: Q5 vs. Q1, OR = 0.958, 95%CI = 0.876–1.048, *P*-trend = 0.3389; HTN: Q5 vs. Q1, OR = 0.928, 95%CI = 0.820–1.050, *P*-trend = 0.3623).

### 3.5. Subgroup Analysis

**Figure 2:**
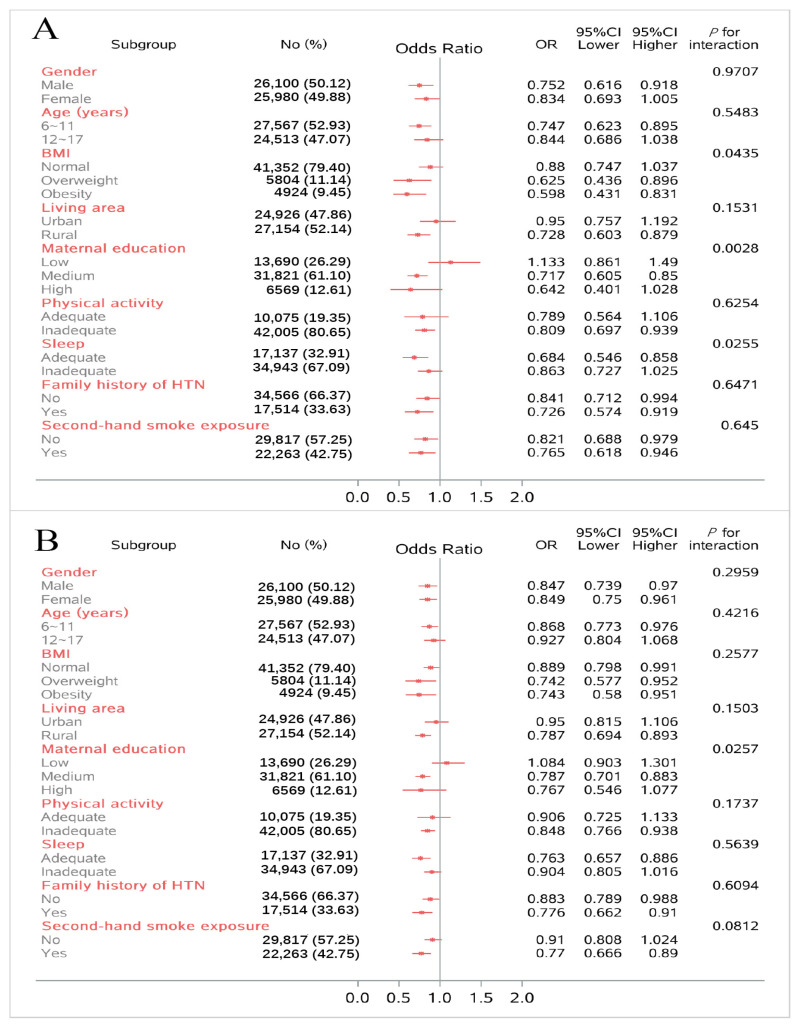
Subgroup analysis of odds ratio (Q5 vs. Q1) between PDP scores and risk of HTN (**A**) and elevated BP (**B**), according to potential risk factors.

Figure 2 shows the forest plots of the subgroup analysis. More details are available in Supplemental Table S4. The protective effects of HTN were observed regardless of second-hand smoking exposure and a family history of hypertension or not. However, for other subgroups, the protective effects of HTN only showed among those who were males, aged 6~11 years old, who were overweight or obese, who lived in the urban area, who had medium educated mothers, and who had inadequate physical activity and adequate sleep behavior. Subgroups observed to have significant results are as follows: BMI, maternal education, and sleep duration.

The protective effects of elevated BP were observed regardless of gender, BMI, and family history of hypertension. For other subgroups, the protective effects of elevated BP only showed among those who were 6~17 years old, lived in rural areas, had medium-educated mothers, had inadequate physical activity and adequate sleep behavior, and who were exposed to second-hand smoke. However, significant results of interaction were only observed in the maternal education subgroup.

## 4. Discussion

In this cross-sectional study, we identified a dietary pattern among Chinese children and adolescents using the RRR method, which is characterized by high intakes of dairy products, mushrooms/edible fungi, fresh vegetables, fresh fruits, fresh eggs, aquatic products, mixed legumes, soybeans and related products, offal, dried fruits, and coarse cereals, with low intakes of refined grains. This dietary pattern score was positively correlated with the intakes of all selected response variables (including dietary fiber, calcium, potassium, phosphorus, and retinol), which were considered protective factors for blood pressure. Meanwhile, this pattern could supply higher amounts of health-related nutrients to children and adolescents during growth and development. Children and adolescents with the highest dietary pattern score had a lower likelihood to have elevated BP and HTN after multiple adjustments compared with those in the lowest quintile. Therefore, this pattern, named the ‘protective dietary pattern’ (PDP) for children’s and adolescents’ BP that we followed with interest, may have potential protective effects on the risk of elevated BP and HTN among children and adolescents.

This study observed significant associations suggesting an advantageous effect of this dietary pattern on the risk of elevated BP and HTN in children and adolescents. The current findings are supported by some previous studies in which dietary patterns characterized by high or low intakes of some of the food groups present in our pattern (e.g., edible fungi and seaweeds, fresh fruits, and vegetables) have been associated with blood pressure [34,35,36]. Ren et al. found that a nut- and algae-less dietary pattern correlated with increased BP levels in children and adolescents compared with children and adolescents with a balanced diet [34]. In Brazilian adolescents with obesity, Neves et al. extracted the ‘Restricted’ pattern characterized by high intakes of low-energy-density foods, such as fresh foods and diet/light products, using the RRR method, and found that the scores of this pattern were inversely associated with SBP and DBP [35]. While Leermakers ETM et al. found that higher adherence to a ‘western-like’ dietary pattern characterized by high intakes of snacks, animal fats, refined grains, confectionery, and sugar-containing beverages was associated with a higher SBP and DBP [36].

The present study discovered that children and adolescents in the highest quintile tend to have lower intakes of carbohydrates, while higher intakes of energy, protein, and micro-nutrients, including those we regarded as response variables. This indicated that children and adolescents who have greater adherence to the protective dietary pattern could be getting energy mainly through fat and protein rather than carbohydrates, which is consistent with the principles of the ketogenic diet to some extent [37,38]. Nevertheless, during the essential period of development, appropriate intake of energy and nutrients and a balanced diet are important for children and adolescents. Further studies are also warranted to test the relationship between different dietary patterns and HTN.

The DASH diet, which has been widely recommended to prevent and treat hypertension, served as a reference tool for assessing the performance of our dietary pattern in this research [39]. Our pattern, like DASH, was represented by higher intakes of fresh fruits and vegetables, dairy products, aquatic products, legumes, and soybeans, and lower intakes of refined grains and red meats. Meanwhile, our pattern also retained extra food groups that have relatively higher loadings, including edible fungi and seaweeds, fresh eggs, and offal. Moore et al. supported the idea that a dietary pattern rich in fruits, vegetables, and dairy products may have beneficial effects on blood pressure change among children and adolescents, and that health-promoting dietary habits established at an earlier age have long-term effects on health outcomes [40]. It is noteworthy that the DASH diet was established based on Western populations; it may be suboptimal for the Chinese population. Hence, there is a great necessity to establish a dietary pattern which is suitable for BP control among Chinese children and adolescents, and to discuss it in the prospective study.

In subgroup analyses, an interaction effect modified by BMI and maternal education level was observed. Among many HTN-related risk factors of children and adolescents, adiposity seems less controversial [41,42]. More so than height or weight, BMI has consistently been shown to be a strong independent predictor of HTN [43]. This study observed a more pronounced protective effect of the dietary pattern on HTN among children and adolescents that were overweight or obese. On the one hand, it might be explained by the intentional transition to a healthier diet among children and adolescents that are overweight or obese after their parents realize their excessive weight condition [44]. On the other hand, HTN-related nutrients, such as calcium and potassium, may compensate for obesity-induced vascular damage to some extent [45]. Thus, a potential implication of our finding is that reducing vascular injury in obese children and adolescents through dietary interventions is also a promising line of research, which is more significant in reducing the onset risk of CVD in early adulthood. In addition, the association was observed only in those whose maternal education was medium and above. A previous study found that children and adolescents whose mothers had elementary school or below education were more likely to consume Western fast food [46]. Because mothers are the primary caregivers of children and adolescents, their education level determines household dietary choices and the formation of dietary habits in children and adolescents.

Recently, it has been widely recognized that methods of assessing dietary status that focus on the overall diet rather than dietary components should be developed [47]. Dietary pattern analysis, including a priori methods and a posteriori methods, provides an approach to evaluate the effect of overall diet on health outcomes. The RRR method, a hybrid method of data-driven methods and a priori knowledge of diet–disease relationships can extract dietary patterns that tend to be more associated with the target disease through the mediation variables [15,48]. However, few studies have focused on the association between dietary patterns and HTN among Chinese children and adolescents using the RRR method, which can generate hypotheses about which food components of a dietary pattern are related to HTN. In addition, various methods of diet assessment, including dietary recall, FFQ, and hybrid methods, have been widely used in epidemiological studies to obtain dietary data for individuals [49]. Since the approach used to calculate dietary scores plays a critical role in determining the relationship with risk factors, which method is more appropriate for assessing long-term diet status still seems debatable.

The dietary patterns identified here are similar to those reported in other samples and are associated with nutrients related to HTN risk. Our results support the use of dietary patterns in guiding public health recommendations for dietary prevention of HTN in children and adolescents. Currently, the limited number of studies focusing on the association of dietary patterns and HTN was almost exclusively based on adult study populations. Only a few studies examined diet patterns in relation to HTN in childhood, with inconsistent findings. However, childhood hypertension has been a considerable and underrecognized public health issue in China and worldwide [50,51]. Given that unmanaged hypertension may lead to vascular dysfunction in early adulthood, it is important to consider dietary intervention strategies that offer lasting cardiovascular benefits to at-risk children and adolescents [52]. Therefore, our study regarding the association of dietary pattern with hypertension and elevated BP is of great significance among Chinese children and adolescents, as it provides new insights into the prevention of hypertension in children and adolescents.

To the best of our knowledge, this is the first study using the RRR method to derive BP-related dietary patterns among Chinese children and adolescents based on a nationally representative sample. The current study has identified a dietary pattern that may have protective effects on blood pressure among Chinese children and adolescents and examined the relationship of this pattern with hypertension in various subgroups to make the results more reliable. Similar studies have been conducted with adult individuals [18]. However, our study has several limitations. First, the causality between the dietary pattern and HTN cannot be confirmed due to the cross-sectional design. Second, our dietary information was collected based on the FFQ; measurement errors may be introduced via recall bias. Third, edible oil and other condiments were not included in dietary assessment in the current study, which may result in underestimation of some nutrient intakes. Fourth, some potential confounders cannot be taken into consideration due to the limited manpower and material resources of large-scale investigations. Fifth, our BP data were based on three averaged BP measurements, taken during a single visit instead of across more than three different occasions. Sixth, we did not include information on supplements and medication use due to the limited data.

## 5. Conclusions

The current study has identified a protective dietary pattern for children’s and adolescents’ blood pressure using the RRR method. Greater adherence to this pattern was associated with lower odds of elevated BP and HTN among Chinese children and adolescents. Our findings supported that protective dietary patterns could serve as a potential approach for the prevention and management of elevated BP and HTN among Chinese children and adolescents.

## Figures and Tables

**Table 1:** Characteristics of participants by quintiles of PDP scores.

Variables	Total (n, %)	Quintile				
		Q1	Q2	Q3	Q4	Q5
Gender *						
Male	26,100 (50.1)	5622 (54.0)	5234 (50.3)	5092 (48.9)	5156 (49.5)	4996 (48.0)
Female	25,980 (49.9)	4794 (46.0)	5182 (49.8)	5324 (51.1)	5260 (50.5)	5420 (52.0)
Age (years) *						
6~11	27,567 (53.0)	5156 (49.5)	5787 (55.6)	5767 (55.4)	5667(54.4)	5190 (49.8)
12~17	24,513 (47.0)	5260 (50.5)	4629 (44.4)	4649 (44.6)	4749 (45.6)	5226 (50.2)
BMI *						
Normal	41,352 (79.4)	8738 (83.9)	8493 (81.5)	8326 (79.9)	7950 (76.3)	7845 (75.3)
Overweight	5804 (11.1)	984 (9.5)	1039 (10.0)	1091 (10.5)	1293 (12.4)	1397 (13.4)
Obese	4924 (9.5)	694 (6.7)	884 (8.5)	999 (9.6)	1173 (11.3)	1174 (11.3)
Living area *						
Urban	24,926 (47.9)	2785 (26.7)	3920 (37.6)	4928 (47.3)	6119 (58.8)	7174 (68.9)
Rural	27,154 (52.1)	7631 (73.3)	6496 (62.4)	5488 (52.7)	4297 (41.3)	3242 (31.1)
Geographical region *						
East	18,909 (36.3)	2034 (19.5)	3052 (29.3)	3814 (36.6)	4599 (44.2)	5410 (51.9)
Central	16,553 (31.8)	3728 (35.8)	3514 (33.7)	3352 (32.2)	3051 (29.3)	2908 (27.9)
West	16,618 (31.9)	4654 (44.7)	3850 (37.0)	3250 (31.2)	2766 (26.6)	2098 (20.1)
Maternal education *						
Primary school or below	13,690 (26.3)	4149 (39.8)	3254 (31.2)	2558 (24.6)	2046 (19.6)	1683 (16.2)
Junior middle school	31,821 (61.1)	5799 (55.7)	6344 (60.9)	6589 (63.3)	6599 (63.4)	6490 (62.3)
High school or higher	6569 (12.6)	468 (4.5)	818 (7.9)	1269 (12.2)	1771 (17.0)	2243 (21.5)
Household income *						
Not given	32,691 (62.8)	6674 (64.1)	6416 (61.6)	6443 (61.9)	6485 (62.3)	6673 (64.1)
Low	7538 (14.5)	1906 (18.3)	1770 (17.0)	1474 (14.2)	1265 (12.1)	1123 (10.8)
Medium	8618 (16.6)	1584 (15.2)	1786 (17.2)	1866 (17.9)	1774 (17.0)	1608 (15.4)
High	323 (6.2)	252 (2.4)	444 (4.3)	633 (6.1)	892 (8.6)	1012 (9.7)
Physical activity *						
Adequate	42,005 (80.7)	1519 (14.6)	1974 (19.0)	2003 (19.2)	2207 (21.2)	2372 (22.8)
Inadequate	10,075 (19.4)	8897 (85.4)	8442 (81.1)	8413 (80.8)	8209 (78.8)	8044 (77.2)
Sedentary behavior (h) *						
<2	7337 (14.1)	1704 (16.4)	1533 (14.7)	1504 (14.4)	1342 (12.9)	1254 (12.0)
2~3	13,242 (25.4)	2684 (25.8)	2667 (25.6)	2716 (26.1)	2659 (25.5)	2516 (24.2)
≥4	31,501 (60.5)	6028 (57.9)	6216 (59.7)	6196 (59.5)	6415 (61.6)	6646 (63.8)
Sleep duration *						
Adequate	34,943 (67.1)	3755 (36.1)	3741 (35.9)	3537 (34.0)	3247 (31.2)	2857 (27.4)
Inadequate	17,137 (32.9)	6661 (64.0)	6675 (64.1)	6879 (66.0)	7169 (68.8)	7559 (72.6)
Family history of HTN *						
No	34,566 (66.4)	7612 (73.1)	7278 (69.9)	6835 (65.6)	6486 (62.3)	6355 (61.0)
Yes	17,514 (33.6)	2804 (26.9)	3138 (30.1)	3581 (34.4)	3930 (37.7)	4061 (39.0)
**Second-hand smoking exposure**						
No	29,817 (57.3)	6077 (58.3)	5979 (57.4)	5973 (57.3)	5833 (56.0)	5955 (57.2)
Yes	22,263 (42.8)	4339 (41.7)	4437 (42.6)	4443 (42.7)	4583 (44.0)	4461 (42.8)

Note: * indicates *p* < 0.0001 (second-hand smoking exposure, *p* = 0.0182). Categorical variables were described as amounts with percentages. Values of polytomous variables may not sum to 100% because of rounding. Abbreviation: BMI, body mass index; HTN, hypertension.

**Table 2:** Association between PDP and DASH scores and risk of blood pressure outcomes in the children and adolescents aged 6~17 years old from the CNHS (2015–2017).

Dietary Pattern	Blood Pressure Outcomes	Quintile	N	No. of Cases	OR (95%CI)		
					Crude Model	Model I	Model II
PDP	Elevated BP	Q1	10,416	1380	†	Reference	Reference
		Q2	10,416	1323		0.903 (0.832, 0.98)	0.894 (0.822, 0.972)
		Q3	10,416	1341		0.883 (0.813, 0.958)	0.878 (0.806, 0.957)
		Q4	10,416	1295		0.881 (0.812, 0.956)	0.885 (0.810, 0.966)
		Q5	10,416	1274		0.838 (0.772, 0.91)	0.849 (0.755, 0.931)
		P-trend				<0.0001	0.0114
	HTN	Q1	10,416	666	†	Reference	Reference
		Q2	10,416	648		0.939 (0.84, 1.051)	0.909 (0.81, 1.02)
		Q3	10,416	638		0.904 (0.808, 1.012)	0.876 (0.779, 0.986)
		Q4	10,416	649		0.888 (0.793, 0.994)	0.882 (0.781, 0.996)
		Q5	10,416	585		0.79 (0.703, 0.887)	0.795 (0.694, 0.911)
		P-trend				<0.0001	0.0018
DASH	Elevated BP	Q1	10,128	1295	†	Reference	Reference
		Q2	13,079	1690		0.979 (0.906, 1.059)	0.991 (0.915, 1.073)
		Q3	9660	1184		0.912 (0.838, 0.993)	0.94 (0.861, 1.025)
		Q4	8059	1036		0.943 (0.863, 1.03)	0.983 (0.897, 1.078)
		Q5	11,154	1408		0.899 (0.828, 0.975)	0.958 (0.876, 1.048)
		P-trend				0.0055	0.3389
	HTN	Q1	10,128	639	†	Reference	Reference
		Q2	13,079	802		0.933 (0.838, 1.039)	0.944 (0.846, 1.053)
		Q3	9660	561		0.871 (0.775, 0.980)	0.902 (0.799, 1.018)
		Q4	8059	508		0.927 (0.821, 1.046)	0.973 (0.858, 1.104)
		Q5	11,154	676		0.861 (0.769, 0.964)	0.928 (0.820, 1.050)
		P-trend				0.0135	0.3623

Note: † Elevated blood pressure (BP) and hypertension (HTN) in children and adolescents are defined as BP between the 90th and 95th percentile, and greater than the 95th percentile, respectively, standardized for gender, age, and height. Therefore, the crude model results for elevated BP and hypertension are left blank in Table 2. Model I: adjusted for age, gender, height, and BMI; Model II: further adjusted for living area (urban and rural), geographic region (east, west, and central), maternal education level, household income, physical activity, sedentary behavior, sleeping time, family history of HTN, second-hand smoking exposure, daily sodium intake (mg/d), and daily energy intake (kcal/d). Abbreviation: PDP, protective dietary pattern; DASH, Dietary Approaches to Stop Hypertension; BP, blood pressure; HTN, hypertension; OR, odds ratio; CI, confidence interval.

## Data Availability

According to the policy of the National Institute for Nutrition and Health, China CDC, data related to this research are not allowed to be disclosed.

## Supplementary Materials

The following supporting information can be downloaded at: https://www.mdpi.com/article/10.3390/nu15234927/s1, Table S1: Corresponding food items and factor loadings of different food groups; Table S2: Correlation coefficients between explanatory variables (absolute factor loadings ≥0.1), response variables applied in the RRR model, and protective dietary pattern among Children from the CNHS 2015–2017; Table S3: Food group intakes of participants in the CNHS 2015–2017 according to quintiles of protective dietary pattern scores; Table S4: Dietary nutrient intake of participants in the CNHS 2015–2017 according to quintiles of protective dietary pattern scores; Table S5: Subgroup analysis of associations between protective dietary pattern scores and risk of HTN; Table S6: Subgroup analysis of associations between protective dietary pattern scores and risk of elevated BP.
