# Supplementary material for: Association of the Protective Dietary Pattern for Blood Pressure with Elevated Blood Pressure and Hypertension among Chinese Children and Adolescents Aged 6–17 Years Old: Data from China Nutrition and Health Surveillance (2015–2017)
Source: Nutrients. 2023 Nov 26;15(23):4927. doi: 10.3390/nu15234927 (PMC10708381; doi:10.3390/nu15234927)
Supplement: Supplementary file 1 [file nutrients-15-04927-s001.zip › nutrients-2737189-supplementary.pdf]

Supplemental Table S1. Corresponding food items and factor loadings of different food groups

Food groups	Contained food items	Factor loadings
Dairy products	Whole milk, low-fat/skim milk, whole milk powder, low-fat milk powder, yogurt, cheese, etc.	0.481796
Edible fungi and seaweeds	Mushrooms, fungus, laminaria japonica, laver, and related products	0.355273
Fresh vegetables	All kinds of fresh vegetables	0.326365
Fresh fruits	All kinds of fresh fruits	0.247939
Fresh eggs	Eggs, duck eggs, goose eggs, quail eggs, etc.	0.242195
Aquatic products	Marine fish, freshwater fish, shrimps, crabs, mollusc, etc.	0.238466
Mixed legumes	Mung beans, red beans, pinto beans, etc.	0.200796
Soybeans and related products	Soya, green soya beans, black soya beans, and related products (soya milk, bean curd, bean curd jelly, bean curd shreds, etc.)	0.191077
Offal	All kinds of organs of animals that are eaten as food	0.140081
Dried fruits	Dried jujube, grapes, persimmon, bananas, apricot, and related products (Without processed)	0.122882
Coarse cereals	Corn, buckwheat, millet, and related products	0.116265
Nuts and seeds	Peanuts, sunflower seeds, walnuts, cashews, pistachios, hazelnuts, etc.	0.091459
Poultries	All kinds of poultries	0.077377
Processed eggs	Salted preserved eggs and preserved eggs (pidan)	0.065108
Tubers	Potatoes, taro, sweet potatoes, etc.	0.061014
Vegetable and fruit juice	Pure vegetable and (or) fruit juice	0.05682
Dried vegetables/pickles	All kinds of dried vegetables, Chinese sauerkraut, pickled Chinese cabbage, pickles, etc.	0.035171
Processed meats	Sausages, ham sausages, luncheon meat, etc.	0.027063
Fried staples	Fried dough sticks, fried dough cakes, instant noodles, and related products	-0.021269
Sweetened beverages	Carbonated drinks, tea drinks, milk drinks, vegetable protein drinks, energy drinks, coffee, fruit and vegetable juice beverages, etc.	-0.022341
Bakery products	Bread, biscuits, cookies, pastries, etc.	-0.036859
Snacks	Potato chips and other puffed foods, preserved/candied fruit, chocolates, candies, ice cream	-0.045811
Beef, lamb and other meats	Beef, lamb, donkey meat, horsemeat, rabbit meat, dog meat, etc.	-0.052031
Refined grains	Rice, wheat flour, and related products	-0.445471

Supplemental Table S2. Correlation coefficients between explanatory variables (absolute factor loadings ≥0.1), response variables applied in RRR model, and protective dietary pattern among Children from CNHS 2015–2017

	**Response variables**					**Protective dietary pattern scores**
	**Fiber**	**Potassium**	**Retinol**	**Calcium**	**Phosphorus**	
**Explanatory variable**						
**Positive factor**						
Dairy products	0.533	0.516	0.264	0.653	0.354	0.501
Edible fungi and seaweeds	0.413	0.471	0.152	0.260	0.376	0.409
Fresh vegetables	0.296	0.389	0.068	0.402	0.322	0.240
Fresh fruits	0.384	0.404	0.170	0.348	0.226	0.354
Fresh eggs	0.136	0.176	0.787	0.207	0.334	0.262
Aquatic products	0.151	0.273	0.240	0.274	0.401	0.366
Mix legumes	0.221	0.344	0.086	0.208	0.263	0.299
Soybeans and related products	0.175	0.243	0.099	0.260	0.296	0.272
Offal	0.071	0.095	0.363	0.082	0.114	0.201
Dried fruits	0.223	0.207	0.072	0.192	0.168	0.281
Coarse cereals	0.281	0.250	0.120	0.201	0.330	0.307
**Negative factor**						
Refined grains	-0.518	-0.604	-0.333	-0.580	-0.437	-0.699
**Response variables**						
Fiber	1.000					
Potassium	0.855	1.000				
Retinol	0.097	0.263	1.000			
Calcium	0.559	0.743	0.389	1.000		
Phosphorus	0.446	0.697	0.508	0.732	1.000	
**Protective Dietary pattern scores**	0.658	0.788	0.450	0.740	0.710	1.000

Note: All P of the correlation coefficients were < 0.0001, except for the correlation coefficients between vegetable and retinol (P = 0.2176).

All the above explanatory variables and response variables were included in the correlation model after energy adjustment (g/1000 kcal or mg/1000 kcal) .

Supplemental Table S3. Food group intakes of participants in CNHS 2015–2017 according to quintiles of protective dietary pattern scores

Food groups (g/1000 kcal)	Total	Q1	Q2	Q3	Q4	Q5
Dairy products	59.2 (15.3,112.4)	8.3 (0,37.3)	42.7 (8,83.1)	70.1 (27.7,118.9)	90 (45.1,137.9)	101.5 (55.3,160.9)
Edible fungi and seaweeds	6.1 (0,17.9)	0.5 (0,6)	3.6 (0,11.7)	6.4 (0.6,16.6)	9.9 (2.7,23.4)	16 (5.3,37.3)
Fresh vegetables	71.9 (38.6,124.1)	48.2 (24.1,84)	68.5 (38.4,116.3)	76.7 (42.9,127)	86.5 (48,143)	88.4 (47.6,154.6)
Fresh fruits	52.4 (26.6,91.4)	26.3 (11.4,48.3)	46.7 (25,78.9)	59.9 (33,99.2)	69.2 (38.6,110.9)	69.7 (39.4,113.4)
Fresh eggs	14.7 (6.2,25.4)	6.9 (0,14.6)	14 (6.2,25.3)	17.7 (8.2,30)	18.7 (9.4,29.7)	17.1 (9.4,25.4)
Aquatic products	4.2 (0,12.9)	0 (0,4.6)	2.5 (0,8.5)	4.3 (0,12.2)	6.8 (1.1,17.5)	10.8 (2.9,24.9)
Mixed legumes	0 (0,0.8)	0 (0,0)	0 (0,0)	0 (0,0.7)	0 (0,1.6)	0 (0,2.9)
Soybeans and related products	2.2 (0.5,5.3)	1 (0,2.9)	1.8 (0.3,4.4)	2.3 (0.6,5.3)	2.9 (0.9,6.3)	3.6 (1.3,7.9)
Offal	0 (0,0)	0 (0,0)	0 (0,0)	0 (0,0)	0 (0,0)	0 (0,0.3)
Dried fruits	0 (0,1)	0 (0,0)	0 (0,0)	0 (0,0.9)	0 (0,2.1)	0.1 (0,3.7)
Coarse cereals	0 (0,5.8)	0 (0,0)	0 (0,3)	0 (0,6.7)	1.1 (0,8.7)	2.7 (0,10.7)
Nuts and seeds	0.9 (0,5.4)	0 (0,2.3)	0 (0,4.2)	1 (0,5.5)	1.9 (0,6.7)	2.9 (0,8.1)
Poultries	3.4 (0,9.1)	1.1 (0,5.2)	2.9 (0,8.4)	3.9 (0,9.9)	4.5 (0,10.8)	4.9 (0.7,11.5)
Processed eggs	0 (0,0)	0 (0,0)	0 (0,0)	0 (0,0)	0 (0,0)	0 (0,1.9)
Tubers	8.6 (1.1,20.4)	5.9 (0,16.7)	9 (0,21.2)	9.4 (1.9,21.5)	9.7 (2.6,21.8)	9.1 (2.6,20.9)
Vegetable and fruit juice	0 (0,0)	0 (0,0)	0 (0,0)	0 (0,0)	0 (0,0)	0 (0,2.5)
Dried vegetables/pickles	0 (0,1.7)	0 (0,0.9)	0 (0,1.7)	0 (0,1.8)	0 (0,1.8)	0 (0,2)
Processed meats	0.9 (0,7.2)	0 (0,3.6)	0 (0,7.2)	1.4 (0,8.4)	2.2 (0,8.7)	2.3 (0,8.2)
Fried staples	0 (0,1.8)	0 (0,0)	0 (0,1)	0 (0,2.2)	0 (0,2.8)	0 (0,2.9)
Sweetened beverages	18.5 (0,62.1)	4.2 (0,40.4)	15.3 (0,57.2)	20.7 (0,65.1)	25.3 (0,71.6)	27.7 (0,73.4)
Bakery products	14.6 (4.6,28.7)	7.7 (0,19.9)	14.1 (3.8,28.9)	16.2 (5.8,30.8)	17.5 (7.5,31.8)	17.4 (7.8,30.2)
Snacks	3.7 (0,11.6)	1.7 (0,8.9)	3.6 (0,12.2)	4.2 (0,12.6)	4.4 (0,12.4)	4.4 (0.3,11.9)
Beef, lamb and other meats	25.4 (12.4,45.2)	23.9 (10.3,46.8)	26.6 (12.9,49.1)	25.7 (12.7,45.5)	25.8 (13,43.9)	24.7 (12.9,41.6)
Refined grains	106.9 (72.8,147.7)	179.1 (149.1,206.9)	129.1 (102.7,154.9)	102.2 (77.6,127)	84.3 (61.1,108.7)	67.1 (46.8,90.1)

Note: Except for beef, lamb and other meats intakes (*P*-trend = 0.1305), all the *P*-trend of the remaining food group intakes in different quintiles of dietary pattern scores were < 0.0001.

Supplemental Table S4. Dietary nutrient intakes of participants in CNHS 2015–2017 according to quintiles of protective dietary pattern scores

Nutrients	Total	Q1	Q2	Q3	Q4	Q5
Energy, kcal	1881.1 (1388.1,2528.1)	1761.3 (1281,2455.1)	1545.3 (1143.3,2094.2)	1664.9 (1270.5,2185)	1931.6 (1533.9,2479.1)	2526.9 (2011.6,3140.1)
Protein, g/1000 kcal	35.7 (31.7,40.6)	31.6 (28.8,34.7)	34.9 (31.4,38.9)	36.3 (32.3,41)	37.4 (33.3,42.2)	39.6 (35.3,44.9)
Fat, g/1000 kcal	24.1 (18.6,30.1)	18.9 (13.7,25.1)	23.6 (18.4,30)	24.9 (19.7,30.7)	25.4 (20.5,30.8)	26.4 (21.5,31.8)
Carbohydrate, g/1000 kcal	168 (152,182.6)	180.8 (165.5,193.4)	169 (153.2,182.4)	166.3 (150.8,180)	164.9 (150,178.5)	160.7 (145.3,174.8)
Dietary fiber, g/1000 kcal	7.2 (4.9,10.1)	4 (3.1,5.1)	6.1 (4.7,7.7)	7.8 (6.1,9.9)	9.3 (7.2,11.5)	10.6 (8,13.2)
Cholesterol, mg/1000 kcal	132.4 (80.4,193.7)	78.7 (43.9,121.5)	125.9 (78.7,183.7)	147.5 (94.6,211.5)	157 (103.7,215.7)	157.4 (110.8,212.2)
Vitamin A, μgRAE/1000 kcal	142 (101.5,193.2)	81.2 (58.2,106.6)	127.9 (100,161.5)	152.4 (119.3,193.8)	171.1 (133.2,220.9)	192.9 (147.1,258.3)
Carotene, μg/1000 kcal	749.8 (493.5,1103.9)	445.7 (285.5,659.2)	679.7 (474.7,964.6)	809.1 (576.4,1127)	919.1 (648.5,1282.6)	968.2 (660.9,1406.9)
Retinol, μg/1000 kcal	61.2 (36.8,91.8)	32 (17.9,49.8)	55.5 (35.5,81.6)	67.4 (44,96.9)	74.5 (49.9,104.9)	80.8 (56.9,116.1)
Thiamine, mg/1000 kcal	0.5 (0.5,0.6)	0.5 (0.5,0.6)	0.5 (0.5,0.6)	0.5 (0.5,0.6)	0.5 (0.5,0.6)	0.5 (0.5,0.6)
Riboflavin, mg/1000 kcal	0.5 (0.4,0.6)	0.3 (0.3,0.4)	0.4 (0.4,0.5)	0.5 (0.4,0.6)	0.5 (0.5,0.6)	0.6 (0.5,0.8)
Niacin, mg/1000 kcal	7.3 (6.4,8.4)	6.8 (6.1,7.6)	7.1 (6.3,8.2)	7.3 (6.3,8.4)	7.5 (6.6,8.7)	7.8 (6.7,9.1)
Vitamin C, mg/1000 kcal	43.2 (28.3,61.8)	23.2 (15.3,32.8)	38.2 (27.5,50.3)	47.6 (35,63.2)	54.4 (39.9,73)	58.2 (41.7,80.2)
Vitamin E, mg/1000 kcal	6.9 (5.2,9)	4.3 (3.4,5.4)	6.1 (5,7.6)	7.2 (5.9,8.8)	8 (6.6,9.8)	9.1 (7.5,11.1)
Calcium, mg/1000 kcal	235.5 (176.3,305)	136.5 (106.3,167.3)	200.7 (170.9,238.5)	244.9 (207.1,292.1)	280.3 (236.3,337.2)	328 (271.3,407.6)
Phosphorus, mg/1000 kcal	466 (418.5,525.2)	397.2 (373.7,421.4)	441.6 (413.1,473.3)	473.2 (438.6,514)	501.9 (463.2,547.2)	551.3 (500,615.8)
Potassium, mg/1000 kcal	1120.2 (892.4,1379.3)	742.1 (635.6,848.2)	983.7 (876.7,1099.9)	1164 (1027.5,1310.)	1313 (1151.4,1497)	1502.5 (1295.4,1739.2)
Sodium, mg/1000 kcal	318.5 (222.7,444.8)	198.8 (133,305.4)	290.2 (209.6,414.6)	326.8 (241.8,448.9)	351.9 (269.2,463.4)	402.9 (309,515.9)
Magnesium, mg/1000 kcal	154.3 (133.9,178.1)	124.9 (112.4,138.7)	143.7 (130.2,159.7)	157.2 (141.7,175.6)	168.3 (151.4,188.8)	182.1 (161.9,205.8)
Iron, mg/1000 kcal	9.2 (7.4,11.6)	6.3 (5.5,7.1)	8.1 (7.2,9.2)	9.4 (8.3,11)	10.8 (9.3,12.7)	12.6 (10.7,15.4)
Zinc, mg/1000 kcal	4.8 (4.3,5.4)	4.5 (4,4.8)	4.6 (4.1,5)	4.7 (4.3,5.3)	5 (4.5,5.6)	5.5 (4.9,6.3)
Selenium, μg/1000 kcal	20.7 (17.4,24.9)	16.3 (14.1,18.8)	19.6 (17.1,22.6)	21.4 (18.5,24.9)	22.8 (19.6,26.8)	24.9 (21,30)
Copper, mg/1000 kcal	1.4 (1.1,1.8)	0.9 (0.8,1.1)	1.2 (1,1.5)	1.5 (1.2,1.8)	1.7 (1.4,2)	1.9 (1.5,2.2)
Manganese, mg/1000 kcal	1.5 (1.2,1.9)	1 (0.8,1.2)	1.3 (1.1,1.6)	1.6 (1.3,1.8)	1.8 (1.5,2.1)	2 (1.7,2.4)

Note: All the P-trend of the above nutrient intakes in different DP quintiles were < 0.0001.

Supplemental Table S5. Subgroup analysis of associations between protective dietary pattern scores and risk of HTN.

Subgroup	N (%)	Quintiles, OR (95% CI)					*P* for interaction
		Q1	Q2	Q3	Q4	Q5	
**Gender**							
Male	26100 (50.12%)	**ref**	0.89 (0.752,1.054)	0.822 (0.69,0.98)	0.824 (0.689,0.986)	0.752 (0.616,0.918)	0.9707
Female	25980 (49.88%)	**ref**	0.922 (0.787,1.08)	0.92 (0.784,1.08)	0.931 (0.788,1.099)	0.834 (0.693,1.005)	
**Age**							
6~11	27567 (52.93%)	**ref**	0.939 (0.811,1.087)	0.878 (0.755,1.021)	0.823 (0.702,0.965)	0.747 (0.623,0.895)	0.5483
12~17	24513 (47.07%)	**ref**	0.862 (0.714,1.04)	0.871 (0.721,1.053)	0.965 (0.798,1.167)	0.844 (0.686,1.038)	
**BMI**							
Normal	41352 (79.4%)	**ref**	0.936 (0.817,1.074)	0.957 (0.832,1.1)	0.945 (0.816,1.095)	0.88 (0.747,1.037)	0.0435
Overweight	5804 (11.14%)	**ref**	0.941 (0.692,1.279)	0.773 (0.56,1.066)	0.74 (0.535,1.024)	0.625 (0.436,0.896)	
Obesity	4924 (9.45%)	**ref**	0.75 (0.555,1.015)	0.646 (0.477,0.876)	0.731 (0.543,0.985)	0.598 (0.431,0.831)	
**Living area**							
Urban	24926 (47.86%)	**ref**	0.962 (0.765,1.211)	0.989 (0.793,1.233)	1.066 (0.86,1.322)	0.95 (0.757,1.192)	0.1531
Rural	27154 (52.14%)	**ref**	0.912 (0.797,1.044)	0.851 (0.737,0.982)	0.78 (0.665,0.915)	0.728 (0.603,0.879)	
**Maternal education**							
Low	13690 (26.29%)	**ref**	0.994 (0.81,1.22)	0.965 (0.773,1.206)	1.23 (0.976,1.55)	1.133 (0.861,1.49)	0.0028
Medium	31821 (61.1%)	**ref**	0.817 (0.705,0.947)	0.837 (0.723,0.97)	0.796 (0.683,0.927)	0.717 (0.605,0.85)	
High	6569 (12.61%)	**ref**	1.263 (0.793,2.012)	0.769 (0.482,1.225)	0.697 (0.442,1.098)	0.642 (0.401,1.028)	
**Physical activity**							
Adequate	10075 (19.35%)	**ref**	1.054 (0.788,1.41)	0.96 (0.711,1.296)	1.024 (0.756,1.386)	0.789 (0.564,1.106)	0.6254
Inadequate	42005 (80.65%)	**ref**	0.882 (0.777,1)	0.862 (0.758,0.981)	0.858 (0.75,0.98)	0.809 (0.697,0.939)	
**Sleep**							
Adequate	34943 (67.09%)	**ref**	0.976 (0.823,1.157)	0.774 (0.644,0.93)	0.838 (0.693,1.015)	0.684 (0.546,0.858)	0.0255
Inadequate	17137 (32.91%)	**ref**	0.862 (0.736,1.009)	0.957 (0.82,1.117)	0.919 (0.783,1.077)	0.863 (0.727,1.025)	
**Family History of HTN**							
No	34566 (66.37%)	**ref**	0.903 (0.787,1.036)	0.866 (0.75,0.999)	0.875 (0.754,1.017)	0.841 (0.712,0.994)	0.6471
Yes	17514 (33.63%)	**ref**	0.921 (0.744,1.14)	0.892 (0.723,1.101)	0.893 (0.722,1.104)	0.726 (0.574,0.919)	
**Second-hand smoking**							
No	29817 (57.25%)	**ref**	0.908 (0.781,1.056)	0.918 (0.788,1.069)	0.897 (0.765,1.052)	0.821 (0.688,0.979)	0.645
Yes	22263 (42.75%)	**ref**	0.918 (0.767,1.099)	0.823 (0.683,0.991)	0.867 (0.718,1.048)	0.765 (0.618,0.946)	

Note: Adjusted for all the potential covariables according to Model II (adjusted for age, gender, BMI, living area, geographic region, maternal education level, household income, physical activity, sedentary behavior, sleeping time, family history of HTN, second-hand smoking, daily sodium intake (mg/d) and daily energy intake (kcal/d).

Abbreviations: HTN, hypertension; ref, reference; OR, odds ratio; 95% CI, 95% confidence interval, BMI body mass index.

Supplemental Table S6. Subgroup analysis of associations between protective dietary pattern scores and risk of elevated BP.

Subgroup	N (%)	Quintiles, OR (95% CI)					*P* for interaction
		Q1	Q2	Q3	Q4	Q5	
**Gender**							
Male	26100 (50.12%)	**ref**	0.9 (0.797,1.016)	0.868 (0.765,0.985)	0.925 (0.813,1.051)	0.847 (0.739,0.97)	0.2959
Female	25980 (49.88%)	**ref**	0.884 (0.787,0.993)	0.882 (0.784,0.992)	0.849 (0.752,0.959)	0.849 (0.75,0.961)	
**Age**							
6~11	27567 (52.93%)	**ref**	0.902 (0.806,1.01)	0.863 (0.77,0.969)	0.868 (0.773,0.976)	0.868 (0.773,0.976)	0.4216
12~17	24513 (47.07%)	**ref**	0.886 (0.781,1.005)	0.897 (0.788,1.022)	0.904 (0.789,1.035)	0.927 (0.804,1.068)	
**BMI**							
Normal	41352 (79.4%)	**ref**	0.916 (0.832,1.01)	0.886 (0.801,0.979)	0.927 (0.837,1.028)	0.889 (0.798,0.991)	
Overweight	5804 (11.14%)	**ref**	0.919 (0.726,1.162)	0.821 (0.645,1.046)	0.757 (0.592,0.969)	0.742 (0.577,0.952)	0.2577
Obesity	4924 (9.45%)	**ref**	0.741 (0.581,0.945)	0.856 (0.676,1.084)	0.792 (0.622,1.007)	0.743 (0.58,0.951)	
**Living area**							
Urban	24926 (47.86%)	**ref**	0.901 (0.768,1.058)	0.939 (0.804,1.096)	0.995 (0.855,1.157)	0.95 (0.815,1.106)	0.1503
Rural	27154 (52.14%)	**ref**	0.898 (0.812,0.992)	0.856 (0.77,0.953)	0.816 (0.727,0.917)	0.787 (0.694,0.893)	
**Maternal education**							
Low	13690 (26.29%)	**ref**	0.955 (0.826,1.103)	0.951 (0.811,1.114)	1.119 (0.948,1.322)	1.084 (0.903,1.301)	
Medium	31821 (61.1%)	**ref**	0.835 (0.749,0.931)	0.826 (0.74,0.921)	0.826 (0.74,0.921)	0.787 (0.701,0.883)	0.0257
High	6569 (12.61%)	**ref**	1.119 (0.782,1.602)	0.974 (0.689,1.378)	0.783 (0.557,1.101)	0.767 (0.546,1.077)	
**Physical activity**							
Adequate	10075 (19.35%)	**ref**	1.083 (0.879,1.334)	0.967 (0.781,1.198)	1.055 (0.85,1.309)	0.906 (0.725,1.133)	0.1737
Inadequate	42005 (80.65%)	**ref**	0.858 (0.782,0.941)	0.862 (0.785,0.948)	0.862 (0.785,0.948)	0.848 (0.766,0.938)	
**Sleep**							
Adequate	34943 (67.09%)	**ref**	0.863 (0.754,0.986)	0.833 (0.726,0.957)	0.847 (0.735,0.977)	0.763 (0.657,0.886)	0.5639
Inadequate	17137 (32.91%)	**ref**	0.914 (0.82,1.018)	0.91 (0.815,1.016)	0.91 (0.813,1.019)	0.904 (0.805,1.016)	
**Family History of HTN**							
No	34566 (66.37%)	**ref**	0.934 (0.844,1.032)	0.913 (0.822,1.013)	0.925 (0.83,1.031)	0.883 (0.789,0.988)	0.6094
Yes	17514 (33.63%)	**ref**	0.883 (0.789,0.988)	0.798 (0.685,0.931)	0.804 (0.689,0.938)	0.776 (0.662,0.91)	
**Second-hand smoking**							
No	29817 (57.25%)	**ref**	0.914 (0.819,1.02)	0.848 (0.757,0.95)	0.893 (0.796,1.003)	0.91 (0.808,1.024)	0.0812
Yes	22263 (42.75%)	**ref**	0.869 (0.763,0.99)	0.919 (0.806,1.049)	0.877 (0.765,1.005)	0.77 (0.666,0.89)	

Note: Adjusted for all the potential covariables according to Model II (adjusted for age, gender, BMI, living area, geographic region, maternal education level, household income, physical activity, sedentary behavior, sleeping time, family history of HTN, second-hand smoking, daily sodium intake (mg/d) and daily energy intake (kcal/d).

Abbreviations: BP, blood pressure; ref, reference; OR, odds ratio; 95% CI, 95% confidence interval, BMI body mass index.
